# Genetic Variations in *AMPK*, *FOXO3A*, and *POMC* Increase the Risk of Extreme Obesity

**DOI:** 10.1155/2024/3813621

**Published:** 2024-10-24

**Authors:** Cinthia Vila Nova Santana, Luiz Alexandre Viana Magno, Adauto Versiani Ramos, Maria Angélica Rios, Valéria Cristina Sandrim, Luiz Armando De Marco, Débora Marques de Miranda, Marco Aurélio Romano-Silva

**Affiliations:** ^1^Faculdade de Medicina, Universidade Federal de Minas Gerais (UFMG), Belo Horizonte, Brazil; ^2^Escola Bahiana de Medicina e Saúde Pública, Salvador, Brazil; ^3^Programa de Pós-Graduação em Ciências da Saúde (PPGCS), Faculdade Ciências Médicas de Minas Gerais (FCMMG), Belo Horizonte, Brazil; ^4^INCT em Neurotecnologia Responsável (INCT-NeurotecR), Belo Horizonte, Brazil; ^5^Hospital Felício Rocho, Belo Horizonte, Brazil; ^6^Instituto de Biociências Botucatu, Universidade Estadual Paulista Júlio de Mesquita Filho (UNESP), Botucatu, Brazil

**Keywords:** *AMPK*, extreme obesity, *FOXO3A*, *POMC*, single nucleotide variants

## Abstract

**Objective:** Genetic variability significantly impacts metabolism, weight gain, and feeding behaviors, predisposing individuals to obesity. This study explored how variations in key genes related to obesity—*FOXO3A* (forkhead box O3), *AMPK* (protein kinase AMP-activated), and *POMC* (proopiomelanocortin)—are associated with extreme obesity (EOB).

**Methods:** We conducted a case–control study with 251 EOB patients and 212 healthy controls with a body mass index (BMI) of less than 25 kg/m^2^. We genotyped 10 single nucleotide variants (SNVs) using TaqMan-based assays.

**Results:** Four SNVs—rs1536057 in *FOXO3A*, rs103685 in *AMPK*, rs934778, and rs6545975 in *POMC*—were associated with an increased risk of EOB. The strongest association was observed with rs934778 (*POMC*), which had a maximum odds ratio (OR) of 5.26 (95% CI: 2.86–9.09). While these genetic variations are closely linked to EOB, they do not affect serum glucose, triglycerides, HDL, LDL, BMI, or waist circumference.

**Conclusions:** These findings indicate that factors beyond traditional metabolic pathways, potentially related to feeding behavior or hormonal regulation, may also link these genetic variations to obesity. Further research in a larger sample is essential to validate these findings and explore their potential to guide clinical interventions and public health strategies.

## 1. Introduction

In recent years, the prevalence of overweight and obesity has increased dramatically worldwide [1], with the World Health Organization (WHO) estimating that 38% of the global population is currently either overweight or obese [2]. This rapid rise has made obesity a critical public health issue [3]. Extreme obesity (EOB), characterized by a body mass index (BMI) of 40 kg/m^2^ or higher, has a more severe impact on health compared to general obesity and overweight. It results in higher healthcare costs, increased prevalence of comorbidities (such as cardiovascular diseases, type 2 diabetes, and sleep apnea), and a greater number of years lived with disability [4–7].

While environmental factors such as malnutrition and low physical activity are well-known contributors to obesity, the significant fat accumulation seen in EOB seems to be driven by specific genetic factors [8–10]. Nevertheless, the genetic basis of EOB remains largely unidentified, indicating a substantial gap in our knowledge compared with what is known about common obesity.

Potential genetic contributors to EOB include *FOXO3A* (forkhead box O3a), *AMPK* (5′ adenosine monophosphate-activated protein kinase), and *POMC* (proopiomelanocortin). Each of these genes plays a significant role in regulating metabolic pathways related to obesity. *FOXO3A*, a key transcription factor in the forkhead box O (FOXO) family, is essential for maintaining metabolic balance [11]. It regulates energy balance by modulating genes crucial for energy production, especially under conditions of glucose restriction [12, 13]. In addition, *FOXO3A* has been associated with lipid accumulation and adipocyte inflammation by regulating autophagy [14]. It also suppresses the transcription of neuropeptide W (NPW), which is involved in the hypothalamic control of feeding behavior [15].

The *AMPK* gene is another crucial regulator of metabolic pathways linked to obesity. AMPK functions as a key regulator of metabolism by enhancing glucose and fatty acid uptake [16], promoting fatty acid oxidation to reduce fat accumulation, and modulating hypothalamic pathways that control appetite and energy use [17]. Moreover, AMPK activation reduces inflammation in adipose tissue, thereby mitigating obesity-related chronic inflammation [18].

POMC neurons in the hypothalamus produce melanocortin peptides that regulate energy expenditure and reduce food intake, playing a vital role in body weight management [19]. Proper excitation of POMC neurons is essential for effective melanocortin release and the regulation of leptin signaling, which directly impacts energy homeostasis [20]. Dysfunction of the *POMC* gene causes severe early-onset obesity [21, 22].

Given the involvement of these genes in crucial metabolic and behavioral pathways, investigating single nucleotide variants (SNVs) within *FOXO3A*, *AMPK*, and *POMC* can offer valuable insights into the molecular mechanisms driving obesity. SNVs in these genes may result in altered transcriptional activity, disrupting pathways essential for adipogenesis, energy homeostasis, and feed behavior, thus exacerbating the metabolic imbalances associated with obesity. In this study, we examined four SNPs in *FOXO3A* (rs1536057, rs2802292, rs3813498, and rs1935952), four in *AMPK* (rs1442760, rs1036851, rs1348316, and rs11584787), and two in *POMC* (rs934778 and rs6545975) to explore their potential roles in obesity-related traits, aiming to elucidate the genetic factors underlying EOB.

## 2. Methods

### 2.1. Study Design and Subjects

The sample size was determined through a statistical power analysis conducted before the study, aimed at detecting a minimum effect size of 0.15. This analysis was performed using *G* ∗ Power 3.1 [23] with an alpha level of 0.05 (two-tailed), a power (1-*β*) of 0.90, and one degree of freedom (df = 1) for the 2 × 2 contingency table. The results indicated that a minimum of 234 participants per group would be needed. Our final sample included 251 patients with EOB who were eligible for bariatric surgery and 212 healthy controls with a BMI of less than 25 kg/m^2^. The case group consisted of 251 patients meeting the National Institutes of Health (NIH) criteria for bariatric surgery, defined as having a BMI greater than 40 kg/m^2^ or a BMI greater than 35 kg/m^2^ with obesity-related comorbidities such as hypertension and diabetes mellitus. These patients had previously attempted to lose weight through dietary changes and structured physical activity, including low-calorie diets, but did not achieve significant weight loss. The patients were randomly selected over the past 5 years (Table 1) from Hospital Felício Rocho and Hospital Santa Casa de Misericórdia in Belo Horizonte, Brazil. The control group included 212 healthy volunteers (unrelated to the case group) with a BMI of less than 25 kg/m^2^. All participants provided written informed consent, and the study was approved by the Ethics Committee of the Universidade Federal de Minas Gerais (protocol 269/06).

### 2.2. Biochemical Analysis

Metabolic parameters, including fasting glucose, triglyceride, high-density lipoprotein (HDL), and low-density lipoprotein (LDL) levels, were measured from venous blood samples collected after an overnight fast of at least 8 h. These measurements were performed using enzymatic colorimetric methods on fully automated equipment following the manufacturer's instructions (Doles Reagentes, Brazil).

### 2.3. Sample Collection and Genotyping

Genomic DNA was extracted from peripheral blood using a nonenzymatic salting-out method [24]. Genotyping was performed using TaqMan Assays. In brief, 50 ng of DNA was used for real-time polymerase chain reaction (PCR) genotyping of tagSNVs, which were selected from the HapMap database (Table 2). TagSNVs were selected based on a minimum allele frequency of 0.25 in Caucasian populations. The specific probes for each SNV are detailed in Supporting Table S1. PCR genotyping was carried out on a Stratagene Mx3005P system (La Jolla, CA, USA) with the following protocol: initial denaturation at 95°C for 10 min, followed by 50 cycles of 15 s at 95°C for denaturation and 1 min at 60°C for annealing and extension. Fluorescence was measured after each PCR cycle. To ensure the accuracy of the genotyping data, quality control measures included retyping at least 10% of the samples.

### 2.4. Statistical Analysis

Quantitative data such as BMI, waist circumference, and levels of glucose, triglycerides, HDL, and LDL were analyzed using Student's *t*-test in GraphPad Prism. Genetic frequencies were compared using UNPHASED [25]. HAPLOVIEW was employed to evaluate pairwise linkage disequilibrium (LD) matrices among SNVs, assess LD block structure, and test for deviations from Hardy–Weinberg equilibrium (HWE) [26]. The D′ value, indicating the strength of the LD, was calculated according to the parameters established previously [27]. To detect gene–gene interactions, we applied multifactor dimensionality reduction (MDR) software (v. 3.0.2), a method that is particularly effective for identifying interactions in small populations [28]. The most accurate models were identified based on 10-fold cross-validation consistency (CVC) and testing balance accuracy (TBA), which reflects the proportion of subjects correctly classified as patients or controls. All the statistical tests were two-tailed, with a significance threshold set at *p* < 0.05.

## 3. Results

Our study included 251 EOB patients, of which 13.7% were male, averaging 37.4 ± 13.2 years of age, and 86.3% were female, averaging 42.2 ± 11.7 years. The control group consisted of 212 healthy individuals, 33.2% of whom were male with an average age of 59.2 ± 12.9 years. The remaining 66.8% were female, with an average age of 58.2 ± 14.1 years. In the EOB group, we observed deviations in clinical and biochemical parameters. Specifically, average BMI exceeded 40 kg/m^2^, and fasting glucose levels were above 100 mg/dL (Table 1).

The allele frequencies in healthy individuals were consistent with those found in Caucasian populations, except for the *AMPK* rs11584787 and *POMC* rs934778 SNVs. All other SNVs were consistent with the HWE (Table 2).

Although none of the *FOXO3A*, *AMPK*, or *POMC* SNVs were directly linked to the clinical characteristics of EOB patients, at least one SNV from each gene showed different distributions between groups, suggesting their potential as biomarkers for EOB (Table 3).

**Figure 1:**
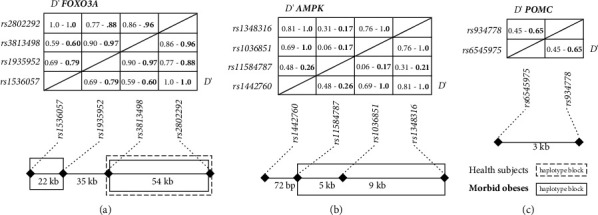
Linkage disequilibrium (LD) analysis. Each square at the top panels (with *D*′ values written within the box) represents a pairwise LD relationship between two SNVs of the (a) *FOXO3A*, (b) *AMPK*, or (c) *POMC* genes. LD values for controls are at the left side of each square, whereas those from EOB are at the right and highlighted in bold. At the bottom, there are the intermarker distance and haplotype blocks. The dashed and solid lines represent haplotype blocks of controls and EOB, respectively.

For *FOXO3A*, only the rs1536057 variant was significantly associated with EOB (Table 3). The “TC” genotype was more prevalent among the EOB group (51.8%) than among the control group (31.4%; *p*=3.4 × 10^−6^), with an odds ratio (OR) of 2.43 (95% CI = 1.59–3.70). Conversely, the “CC” genotype was less common in EOB patients (40.6%) than that in healthy controls (59.7%; *p*=0.0001). Consequently, the nonancestral “T” allele was more common in the EOB group, with a frequency of 33.5% (*p*=0.0053). All these associations remained statistically significant after performing 1000 permutation tests. Haplotype analysis was conducted to identify recombination patterns, revealing strong LD between rs1536057 and rs2802292 (*D*′ = 1.0) in both groups. The most frequent haplotype observed was C–T in both EOB patients (49.9%) and healthy controls (51.3%). However, EOB subjects presented a greater prevalence of the “T–G” haplotype (33.5%; *X*^2^=6.13; *p*=0.013) and a lower prevalence of the “C–G” haplotype (16.6%; *X*^2^= 5.68; *p*=0.017) than controls did (25.8% for “T-G” and 23.0% for C-G in controls). In addition, the LD between rs3813498 and rs1935952 was greater in EOB patients (*D*′ = 0.97) than that in healthy individuals (*D*′ = 0.90), leading to the formation of a haplotype block specifically in EOB patients (Figure 1(a)).

In the comparison between the two groups, we observed a heterogeneous distribution of *AMPK* SNVs, with one out of four showing a significant difference (Table 3). The “TT” genotype of rs1036851 was significantly more prevalent in EOB patients (35.4%) than that in healthy subjects (13.9%; *p*=6 × 10^−7^). Conversely, the frequency of the heterozygous “CT” genotype was significantly lower in EOB patients (42.2%) than that in controls (59.4%) (*p* < 0.0001). As a result, the major allele “T” was more common in the EOB group (56.5%), whereas the “C” allele was more common in healthy subjects (56.4%). The significant difference in allele frequency between the groups suggests that carriers of the “C” allele may be less susceptible to EOB, as indicated by OR analysis (OR = 0.60; 95% CI = 0.46–0.79). In addition, we found notable differences in the LD patterns of *AMPK* between the groups. EOB patients exhibited a strong LD block of 14 kb, encompassing rs1442760, rs1036851, and rs1348316 (*D*′ = 1.0), which was not present in healthy subjects (Figure 1(b)). The most common haplotypes within this block were “T–T–A” (43.8%) and “C–C–G” (43.6%) in EOB patients. These *p* values remained significant after 1000 permutation tests, similar to the results observed with *FOXO3A*.

The *POMC* SNVs investigated demonstrated an association with EOB (Table 3). Among these, rs934778 had a significantly lower frequency of the nonancestral “GG” genotype in the EOB group (8.5%) than that in the control group (30.5%; *p*=3.4 × 10^−9^), leading to a greater distribution of the “A” allele in EOB subjects (71.7%) than in controls (52.9%; *p*=6.4 × 10^−9^). These findings suggest that carriers of the “A” allele may have an increased risk of developing EOB (OR = 2.26; 95% CI = 1.71–2.98). Moreover, the rs6545975 SNV had a notably lower frequency of the homozygous “CC” genotype in EOB patients (18.3%) compared to that of controls (28.7%) (*p*=0.0318; OR = 0.53; 95% CI = 0.32–0.88). This finding indicates that carriers of the “C” allele may have a reduced risk of developing EOB (OR = 0.71; 95% CI = 0.54–0.93), as the EOB group presented a lower frequency of the mutant “C” allele (39.6% compared with 47.7% in controls; *p*=0.0128). All significant *p* values were sustained after 1000 permutation tests. However, unlike the other markers, no significant LD pattern was observed between these *POMC* SNVs (Figure 1(c)).

**Figure 2:**
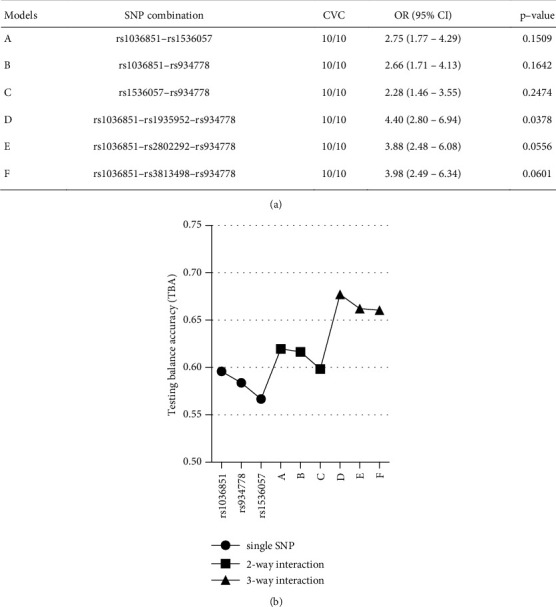
Gene–gene interactions. (a) The best two-and three-marker models (A–F; three of each) were selected by the MDR analyses. CVC: cross-validation consistency; OR = odds ratio. (b) Maximum testing balanced accuracy (TBA) of the best interaction models in comparison with the three single markers associated with EOB identified in this study. A significant *p* value is in bold.

To explore potential interactions between individual markers in EOB, we conducted a gene–gene analysis focusing on two- and three-way combinations. According to the MDR output, the most effective two-marker model involved an interaction between rs1036851 and rs1536057 (Model A) (Figure 2(a)), with a TBA of 0.619 (Figure 2(b)) and a CVC of 10/10. The most effective three-marker models were rs1036851, rs1935952, and rs934778 (Model D), which increased the TBA to 0.677 with a CVC of 10/10. Among these models, only Model D was statistically significant (*X*^2^ = 4.315; *p*=0.0378), with an OR of 4.40 (95% CI = 2.80–6.94).

## 4. Discussion

Extensive research on the genetic basis of obesity has identified specific loci that may either increase or decrease the risk of developing the condition [29, 30]. In this study, we investigated whether certain variations in the *FOXO3A*, *AMPK*, and *POMC* genes contribute to the risk of EOB. Our findings identified significant links between EOB and all three genes, with SNVs rs1536057 (*FOXO3A*), rs1036851 (*AMPK*), and rs934778 and rs6545975 (*POMC*) exhibiting particularly strong effects. Notably, these SNVs have not been previously linked to EOB.

Of the four FOXO3A SNVs analyzed, only rs1536057 showed significant differences in genetic frequency. Interestingly, this SNV has been previously associated with bipolar disorder [31] and tuberculosis [32]. Our analysis suggested that individuals with the TC genotype have a 2.4-fold greater susceptibility to developing EOB. For *AMPK*, the allelic frequency of rs1036851 in EOB patients shows an inverse distribution compared with that in healthy individuals; while “C” is the major allele in healthy subjects, “T” is more frequent in EOB patients. Specifically, the frequency of the “TT” genotype is 2.5 times greater in EOB patients than in controls, suggesting that individuals with this genotype are more susceptible to EOB than those with the “CC” genotype. The *POMC* SNVs (rs934778 and rs6545975) exhibited distinct distributions between the EOB and healthy groups. For rs934778, the nonancestral “T” allele was more prevalent in EOB patients, leading to a higher frequency of the “TT” genotype in this group. This finding is particularly significant, as individuals with the “TT” genotype have the highest risk for EOB (OR = 5.26; 95% CI = 2.88–9.16). Conversely, the nonancestral “C” allele of rs6545975 is associated with a lower likelihood of developing EOB, with ORs of 0.71 (95% CI = 0.55–0.94) under the allelic model and 0.53 (95% CI = 0.33–0.9) under the genotype (“CC”) model.

The significance of these polymorphisms is uncertain because the four SNVs associated with EOB are located in noncoding intronic regions. These SNVs might act as markers for other variants in regulatory regions of the genome that exhibit high LD. For example, the haplotype block (*D*′ = 1) formed exclusively by rs1442760, rs1036851, and rs1348316 in the EOB group spans a genomic region of approximately 15.1 kb, including the last six exons and the 3′ untranslated region (UTR) of the *AMPK* gene. Our haplotype analysis also revealed that the common *FOXO3A* haplotype block rs3813498–rs2802292 was distributed differently between EOB patients and controls. However, the observed ORs (1.33–1.88) were not significantly different from those obtained from the individual marker analyses (0.94–1.29).

*FOXO3A*, *AMPK*, and *POMC* are involved in regulating glucose and lipid metabolism [33–37]. For example, a previous study showed that individuals with the minor “G” allele of rs2802292 in *FOXO3A* exhibited increased insulin sensitivity [38]. However, we did not observe any significant effects of the studied variations on serum glucose, triglyceride, HDL, or LDL levels between the case and control groups. A similar trend was observed for BMI and waist circumference. However, we acknowledge that our results may have been affected by the lack of adjustment for ancestry [39].

Since these polymorphisms do not appear to affect the metabolic profile, it is plausible that they increase the risk of EOB by influencing feeding behavior or inflammation. For example, the expression of a constitutively nuclear mutant FOXO1 in the hypothalamus of rodents has been shown to impair leptin's ability to reduce food intake [40]. Similarly, FOXO3 mediates decreases in hypothalamic *α*-melanocyte-stimulating hormone (*α*-MSH) levels [41]. In addition, *POMC* knockout (KO) mice exhibit greater food intake than their wild-type littermates do [42]. Unfortunately, we did not collect data on food intake in our sample, which could have provided insights into whether these genetic variations influence postprandial inhibitory signaling or the amount of food consumed.

We also emphasize that the *POMC* gene not only influences feeding behavior but also may play a role in obesity-related hypothalamic inflammation. Prolonged obesogenic diets trigger inflammatory responses in the hypothalamus, which are associated with a reduced number of appetite-suppressing *POMC* neurons. For example, hypercaloric diet-induced obese mice accumulate activated microglia within the hypothalamus, leading to local hypersecretion of the proinflammatory cytokine *TNF-α*, altered *POMC* neuron excitability, and increased food intake [43]. Similar hypothalamic damage, associated with inflammatory markers, has also been observed in humans [44, 45]. In light of our findings, further studies are needed to investigate whether the *POMC* variations associated with EOB are linked to broader systemic or hypothalamic inflammation that contributes to the development of EOB.

The present study did not identify any significant epistatic interactions among the three genes contributing to EOB. Two- and three-way interaction models were constructed using all the SNVs. Nevertheless, the most effective model (Model D: rs1036851–rs1935952–rs934778) did not show a significantly greater effect than the individual marker rs934778 alone. This outcome underscores the independent effects of *FOXO3A*, *AMPK*, and *POMC* SNVs in influencing the genetic predisposition to EOB.

The results of this study should be interpreted with caution. First, other obesity-influencing factors such as environmental influences (e.g., physical activity, alcohol consumption, and smoking) and epigenetics were not considered despite their potential impact on EOB genetic risk. Moreover, our study did not cover all possible genetic variations within the targeted genes. Collecting a more comprehensive sample from EOB patients presents significant challenges due to the complexity of the disease. However, it is important to note that positive results were upheld after 1000 permutation tests, indicating a low likelihood of type I errors.

Moreover, the EOB and control groups in our study were not matched for age or sex, which could introduce confounding factors affecting the genetic associations observed. Despite this, the age mismatch between the groups may not significantly impact our findings, as the onset of EOB often occurs earlier in life [46]. Finally, we chose to include all SNVs in our analysis, regardless of their HWE *p* value, to capture potentially meaningful biological phenomena that might otherwise be overlooked.

In summary, the results of this study support our initial hypothesis that genetic variations in *FOXO3A*, *AMPK*, and *POMC* may contribute to susceptibility to EOB. Notably, these polymorphisms did not seem to affect serum levels of glucose, triglycerides, HDL, or LDL in EOB patients. Further investigations are necessary to fully understand the biological significance of these genetic variations. In addition, we recommend replicating these findings in a larger and more diverse sample that accounts for environmental factors to better elucidate the impact of these genetic variants on EOB [47–50].

## Figures and Tables

**Table 1:** Characteristics of extremely obese and healthy individuals included in this study.

	Reference values	Extreme obese (*n* = 251)	Healthy subjects (*n* = 212)	*p* value
Male/fem (%)	—	13.7/86.3	33.2/66.8	—
Age (years)	—	43.3	58.51	—
BMI (kg/m^2^)	< 25.0	47.8 ± 6.8	24.13 ± 2.82	**< 0.0001**
Waist circumference (cm)				
Male	102	136.15 ± 13.48	88.40 ± 9.68	**< 0.0001**
Female	88	122.97 ± 13.23	87.19 ± 9.63	**< 0.0001**
Glucose (mg/dL)	≤ 100	106.89 ± 39.42	79.83 ± 11.84	**< 0.0001**
Triglycerides (mg/dL)	≤ 150	134.02 ± 74.72	141.4 ± 58.89	0.462
HDL (mg/dL)	≥ 40	44.44 ± 8.53	42.42 ± 13.74	0.346
LDL (mg/dL)	≤ 130	125.01 ± 30.24	118.63 ± 28.62	0.176

*Notes:* Reference values according to the Brazilian College of Cardiology. Significant *p* values (*t*-tests) are in bold.

Abbreviations: BMI, body mass index; HDL, high-density lipoprotein; LDL, low-density lipoprotein.

**Table 2:** Position and Hardy–Weinberg equilibrium (HWEpval) of the studied genes.

SNVs	Chromosome position	Gene location	Nucleotide change	HWEpval
*FOXO3A*				
rs1536057	chr.6 108564420	Intron 1	C > T	0.502
rs2802292	chr.6 108587315	Intron 1	G > T	1
rs3813498	chr.6 108622962	Intron 1	T > C	0.426
rs1935952	chr.6 108677702	Intron 2	C > G	0.518
*AMPK*				
rs1442760	chr.1 147156828	UTRa	T > C	0.244
rs1036851	chr.1 147162171	Intron	T > C	1
rs1348316	chr.1 147171976	Intron	G > A	0.119
rs11584787	chr.1 147156900	UTRa	C > G	<0.001
*POMC*				
rs934778	chr.2 25166355	Intron	A > G	<0.001
rs6545975	chr.2 25162616	Intron	T > C	0.001

^a^UTR: untranslated region. Significant *p* values (*X*^2^) are in bold. HWpval (Hardy‒Weinberg equilibrium *p* value) was calculated in the total sample.

**Table 3:** *FOXO3A*, *AMPK*, and *POMC* SNVs in extremely obese (EOB) and healthy individuals.

SNV	Controls (Freq %)	EOB (Freq %)	OR (95% IC)	*χ* ^2^	*p* a	*p*_1000_ b
*FOXO3A* rs1536057						
CC	101 (59.7)	102 (40.6)	1.0	17.15	**0.0001** c	**0.00099**
TC	53 (31.4)	130 (51.8)	2.43 (1.59–3.70)			
TT	15 (8.9)	19 (7.6)	1.25 (0.60–2.60)			
C∗	255 (75.4)	334 (66.5)	1.0	7.76	**0.0053** d	**0.00199**
T	83 (24.6)	168 (33.5)	1.54 (1.13–2.11)			
*FOXO3A* rs2802292						
GG	50 (26.1)	58 (23.3)	1.0	2.29	0.3188^c^	—
TG	88 (45.8)	132 (53.0)	1.29 (0.81–2.06)			
TT	54 (28.1)	59 (23.7)	0.94 (0.55–1.6)			
G∗	188 (49.0)	248 (49.8)	1.0	0.061	0.8044^d^	—
T	196 (51.0)	250 (50.2)	0.97 (0.74–1.26)			
*FOXO3A* rs3813498						
TT	100 (51.8)	122 (48.6)	1.0	0.91	0.6332^c^	—
TC	73 (37.8)	106 (42.2)	1.19 (0.8–1.77)			
CC	20 (10.4)	23 (9.2)	0.94 (0.49–1.81)			
T∗	273 (70.7)	350 (69.7)	1.0	0.10	0.7457^d^	—
C	113 (29.3)	152 (30.3)	1.05 (0.78–1.40)			
*FOXO3A* rs1935952						
CC	66 (37.3)	73 (29.2)	1.0	3.46	0.1768^c^	—
GC	81 (45.8)	135 (54.0)	1.51(0.98–2.32)			
GG	30 (16.9)	42 (16.8)	1.27 (0.71–2.25)			
C∗	213 (60.2)	281 (56.2)	1.0	1.34	0.2467^d^	—
G	141 (39.8)	219 (43.8)	1.18 (0.89–1.55)			
*AMPK* rs1442760						
TT	63 (30.7)	85 (35.3)	1.0	1.22	0.5419^c^	—
TC	93 (45.4)	100 (41.5)	0.79 (0.51–1.21)			
CC	49 (23.9)	56 (23.2)	0.83 (0.50–1.37)			
T∗	219 (53.4)	270 (56.0)	1.0	0.74	0.3885	—
C	191 (46.6)	212 (44.0)	0.89 (0.68–1.15)			
*AMPK* rs1036851						
TT	26 (13.9)	82 (35.4)	1.0	25.64	**<0.0001** c	**0.0009**
CT	111 (59.4)	98 (42.2)	0.29 (0.17–0.48)			
CC	50 (26.7)	52 (22.4)	0.34 (0.19–0.61)			
T∗	163 (43.6)	262 (56.5)	1.0	13.16	**0.0002** d	**0.0009**
C	211 (56.4)	202 (43.5)	0.60 (0.46–0.79)			
*AMPK* rs1348316						
GG	58 (28.4)	71 (29.5)	1.0	2.18	0.3363^c^	—
AG	101 (49.5)	104 (43.1)	0.83 (0.53–1.29)			
AA	45 (22.1)	66 (27.4)	1.17 (0.70–1.96)			
G∗	217 (53.2)	246 (51.0)	1.0	0.31	0.5758^d^	—
A	191 (46.8)	236 (49.0)	1.08 (0.83–1.40)			
*AMPK* rs11584787						
CC	83 (43.9)	109 (45.2)	1.0	1.11	0.5722^c^	—
CG	48 (25.4)	68 (28.2)	1.09 (0.68–1.74)			
GG	58 (30.7)	64 (26.6)	0.84 (0.53–1.32)			
C∗	214 (56.6)	286 (59.3)	1.0	0.68	0.4089^d^	—
G	164 (43.4)	196 (40.7)	0.89 (0.68–1.17)			
*POMC* rs934778						
AA	76 (36.2)	122 (51.9)	1.0	36.27	**<0.0001** c	**0.0009**
AG	70 (33.3)	93 (39.6)	0.83 (0.54–1.26)			
GG	64 (30.5)	20 (8.5)	0.19 (0.11–0.35)			
A∗	222 (52.9)	337 (71.7)	1.0	33.31	**<0.0001** d	**0.0009**
G	198 (47.1)	133 (28.3)	0.45 (0.34–0.59)			
*POMC* rs6545975						
TT	65 (33.3)	94 (39.2)	1.0	6.89	**0.0318** c	**0.0399**
TC	74 (38.0)	102 (42.5)	0.94 (0.61–1.45)			
CC	56 (28.7)	44 (18.3)	0.53 (0.32–0.88)			
T∗	204 (52.3)	290 (60.4)	1.0	6.19	**0.0128** d	**0.0279**
C	186 (47.7)	190 (39.6)	0.71 (0.54–0.93)			

^∗^Ancestral alleles, which were used as a reference for odds ratio (OR) analyses;

^a^ *p* value before 1000 permutations;

^b^ *p* value after 1000 permutations only performed when *p* < 0.05 (highlighted in bold);

^c^genotype association;

^d^allele association.

## Data Availability

The data used to support the findings of this study are available from the corresponding author upon request.

## Supporting Information

Supporting Information Supporting Table S1. The table lists the single nucleotide variants (SNVs), along with their corresponding TaqMan assays and context sequences.
