# Supplementary material for: Genetic Variations in AMPK, FOXO3A, and POMC Increase the Risk of Extreme Obesity
Source: J Obes. 2024 Oct 24;2024:3813621. doi: 10.1155/2024/3813621 (PMC11527528; doi:10.1155/2024/3813621)
Supplement: Supporting Information — Supporting Table S1. The table lists the single nucleotide variants (SNVs), along with their corresponding TaqMan assays and context sequences. [file 3813621.f1.docx]

Supplementary Material

Genetic variations in AMPK, FOXO3A, and POMC increase the risk of extreme obesity

by Santana et al.

Table of Contents

[Supplementary Material 1: Selected SNVs and assay sequences 2](#_Toc174204292)

# Supplementary Material 1: Selected SNVs and assay sequences

| **SNVs** | **TaqMan Assays** | **Context Sequence [VIC/FAM]** |
| --- | --- | --- |
|  | | |
| **FOXO3A (forkhead box O3)** | | |
| rs1536057 | C___8701842_10 | GTCATGATTAGTCATTTTGGAAGCA**[C/T]**GCATGTGCATTTAAACCACACTACT |
| rs2802292 | C__16097219_10 | TGCTCACAAGAGCTCAGGGCTGGGA**[G/T]**AAGCCTCTGTGTGACAGATGAAGGG |
| rs3813498 | C____431204_10 | AAAAAAAAAAAAAGCTGGAAGAGTT**[C/T]**CCCTTCCTGAGCTTCAGTGGAAGCA |
| rs1935952 | C__11957503_10 | TGGTTTATTAAGTGTTATTGTGAGG**[C/G]**GAAAAAACCAAATCCTCTTCCAGAG |
|  | | |
| **AMPK (protein kinase AMP-activated non-catalytic subunit beta 2)** | | |
| rs1442760 | C___8719003_10 | AGATGGTTATACACTGCTAATTCTG**[T/C]**ACAGTGAGATCCAAGGACACCACCA |
| rs1036851 | C___8719006_10 | AGTTCTTTTGTACACCCCCAAATGA**[T/C]**AACTACTCCTTTATGTTGTGCTCAA |
| rs1348316 | C___8719009_30 | TGCAGTACTGACACCAACTGCGGGC**[G/A]**TGGGACGCTTACCTTGGAGTCAGGG |
| rs11584787 | C___1745072_10 | TTTTTAAAAGAGATCATTTTCAGTT**[G/C]**TAAGTTACTCAACCCTTTATTCTAT |
|  | | |
| **POMC (proopiomelanocortin)** | | |
| rs934778 | C___8722915_10 | CAGGCTGTTCCCATTGTACCCTGCC**[A/G]**GTGATCTATTCAAGTACACAGCCAG |
| rs6545975 | C__32192453_10 | TTTTTAAACATATTTTTAATAATGC**[C/T]**TCAACCTGTGTGAACCTGGGAGGTG |
|  |  |  |

The sequence in brackets represents the polymorphic site
